# Cerebrospinal fluid metabolomes of treatment-resistant depression subtypes and ketamine response: a pilot study

**DOI:** 10.1007/s44192-024-00066-5

**Published:** 2024-04-17

**Authors:** Jon Berner, Animesh Acharjee

**Affiliations:** 1 Woodinville Psychiatric Associates, 18500 156Th Ave NE #100, Woodinville, WA 98072 USA; 2 Institute of Cancer and Genomics Sciences, University of Birmingham, Birmingham, UK; 3 Institute of Translational Medicine, University Hospitals Birmingham NHS Foundation Trust, Birmingham, UK; 4 MRC Health Data Research UK (HDR UK), London, UK

## Abstract

Depression is a disorder with variable presentation. Selecting treatments and dose-finding is, therefore, challenging and time-consuming. In addition, novel antidepressants such as ketamine have sparse optimization evidence. Insights obtained from metabolomics may improve the management of patients. The objective of this study was to determine whether compounds in the cerebrospinal fluid (CSF) metabolome correlate with scores on questionnaires and response to medication. We performed a retrospective pilot study to evaluate phenotypic and metabolomic variability in patients with treatment-resistant depression using multivariate data compression algorithms. Twenty-nine patients with treatment-resistant depression provided fasting CSF samples. Over 300 metabolites were analyzed in these samples with liquid chromatography-mass spectrometry. Chart review provided basic demographic information, clinical status with self-reported questionnaires, and response to medication. Of the 300 metabolites analyzed, 151 were present in all CSF samples and used in the analyses. Hypothesis-free multivariate analysis compressed the resultant data set into two dimensions using Principal Component (PC) analysis, accounting for ~ 32% of the variance. PC1 accounted for 16.9% of the variance and strongly correlated with age in one direction and 5-methyltetrahydrofolate, homocarnosine, and depression and anxiety scores in the opposite direction. PC2 accounted for 15.4% of the variance, with one end strongly correlated with autism scores, male gender, and cognitive fatigue scores, and the other end with bipolar diagnosis, lithium use, and ethylmalonate disturbance. This small pilot study suggests that complex treatment-resistant depression can be mapped onto a 2-dimensional pathophysiological domain. The results may have implications for treatment selection for depression subtypes.

**Supplementary Information:**

The online version contains supplementary material available at 10.1007/s44192-024-00066-5.

## Introduction

Depression is one of the most prevalent and disabling mental health conditions in the United States [1]. Depression is a polygenic and multifactorial disorder with a highly variable phenotypic presentation due to multiple gene-environment interactions [2, 3]. Treatments for depression are only modestly effective, and the majority of people with depression experience a relapse at some point in life. Around 30% of patients with depression have treatment-resistant depression, and almost all patients experience substantial barriers to accessing novel and often stigmatized or expensive secondary treatments (e.g., ketamine, buprenorphine, magnetic stimulation, and electroconvulsive therapy) [4]. Non-response to even these secondary treatments, however, remains common in specialty practice. A better understanding of the polygenic determinants of depression may improve the selection of specific treatment combinations, especially given the multitude of possible combinations implied by the increasing number of incrementally effective medications entering clinical practice.

Screening of patients for treatment currently relies on low-tech methods, such as aggregate checklists of self-reported symptoms, which do not provide the precision to minimize sequential empirical trials in the community. Theoretically, tissue biopsy could provide a more accurate alternative to self-reporting. Recent reports describe the use of cerebrospinal fluid (CSF) for the diagnosis of bipolar disorder [5] and the management of treatment-resistant depression [6, 7].

A CSF metabolomic profile, for which over 300 compounds are measured to analyze biochemical processes in the brain, can now be generated at a price ($1500) similar to standard diagnostic practices, imaging across multiple modalities, or serology for autoimmune disease. The rapid development of multivariate data acquisition and statistical analysis methods of such large data sets allows for a shift of attention toward tissue diagnostics. The complex CSF metabolomic profile can be compressed into aggregate statistics with data compression algorithms.

This retrospective pilot study describes the results of simultaneously evaluating phenotypic and metabolomic variability in a select group of patients with treatment-resistant depression using multivariate data compression algorithms. Scores on multiple self-administered questionnaires were obtained from patient files, and CSF samples were obtained from the patients during routine clinical evaluations. The objective of this pilot study was to determine whether compounds in the CSF metabolomic profile correlate, in both univariate and multivariate analyses, with scores on the questionnaires and response to medication treatments, e.g., ketamine.

## Materials and methods

### Patients

Chart review from a subset of 2500 active patients in a community fee-for-service clinic identified 29 patients that had been evaluated with CSF sampling for central folate deficiency [6] or anesthesia sensitivity [7] from 3/2019 to 10/2020 in the context of treatment-resistant depression. These patients had a distinct clinical profile marked by chronic illness, multiple failed medication trials, risk tolerance to stigmatized procedure, intellectual interest in translational techniques, and the financial resources to cover residual procedure costs after insurance. Enrichment for schizophrenia and acute unipolar illness is likely small. There were no individual cases of epilepsy or dementia as a dominant indication, as would be frequently ordered in pediatric neurology centers. Treatment resistance was defined as the lack of effect of treatment with more than two antidepressant trials in superficial unipolar syndromes or of three major classes of mood stabilizers in bipolar syndromes (lithium, anticonvulsant, and antipsychotic).

Standard demographic variables such as age, gender, and body mass index (BMI) were extracted from their charts. Clinical information included scores on multiple self-administered questionnaires: the 9-item Patient Health Questionnaire (PHQ-9), which determines depression severity [8]; the 7-item Generalized Anxiety Disorder (GAD-7), which assesses the severity of generalized anxiety disorder [9]; the World Health Organization Disability Assessment Schedule 2.0 (WHODAS 2.0), a 36-item questionnaire to assess functional disability associated with physical and mental disorders [10, 11]; the Autism-spectrum Quotient (AQ), a 50-item questionnaire to assess the degree of autistic traits [12]; and the 6-item Karolinska Scales of Personality (KSP-6), which identifies patients with depression at high risk of having underlying mitochondrial dysfunction [13]. The KSP-6 is used to estimate brain mitochondrial activity. The six items on the KSP-6 are derived from the original 135-item Karolinska Scales of Personality and were selected to maximally correlate with alpha-ketoglutarate substrate-derived *in-vitro* mitochondrial ATP production in muscle biopsies [13].

Medical chart review also revealed concurrent use of medication (mood stabilizers: lithium and anticonvulsants, benzodiazepines, opioids, stimulants, antidepressants), dominant clinical impressions (bipolar disorder, pain, or depression) and subsequent use of, and response to, ketamine or a combination of ketamine and rapamycin (the combination is hereafter referred to as rapamycin). A bipolar diagnosis is preferred in the context of antidepressant induced mania, preferential response to mood stabilizer relative to unopposed antidepressant trials, or classical episodes of mania/hypomania marked by excessive goal related activity with adverse consequences.

Ketamine treatment consisted of daily oral use, typically before sleep, to minimize functional impairment from maximal delirium 1 h after ingestion. The average dose in our clinic was 4.23 mg/kg oral equivalent. Ketamine response is defined by patient preference for refills more than 1 month after the maximal tolerated dose given, as placebo effect is unlikely at this duration in a chronic population. This protocol is similar to our previous work with intranasal ketamine in 110 consecutive patients with similar substantial comorbidities and polypharmacy [14].

### CSF sampling and metabolomic profiling

CSF was obtained in the morning in an outpatient surgical center by a board-certified anesthesiologist after cessation of nutritional supplements for three days and overnight fasting. Samples were transported overnight on dry ice for analysis.

Metabolomic profiling was performed on the CSF samples using a Metabolon platform with four types of liquid chromatography-mass spectrometry (LC–MS) at a clinical diagnostics laboratory (Baylor Genetics, Houston TX) with extensive expertise in CSF analysis (see for example [15]). An additional CSF sample of each patient was analyzed for absolute levels of 5-methyltetrahydrofolate (5-MTHF), the predominant form of folate in CSF, with liquid chromatography-electrospray tandem mass spectrometry (as described [16]) at another clinical diagnostics laboratory (Institute of Metabolic Disease, Baylor Scott & White Research Institute, Dallas).

### Ethical approval

This retrospective case review was exempt from Institutional Review Board approval under Category 2 of the Basic Policy for Protection of Human Research Subjects Subpart A Sect. 46.101 of the US Department of Health and Human Services [17].

### Data analysis

All metabolomic data were analyzed with hypothesis-free multivariate data reduction methods using R statistical software [18] and the MetaboAnalyst tool [19]. Both supervised and unsupervised methods were used.

The supervised methods (Random Forest Analysis and Canonical Correlation Analysis) used labeled input data; for example, an outcome variable (or response) was considered in the analysis as either responded or not responded. *Random Forest Analysis* [20], a method using binary classification, analyzed ketamine or rapamycin response as outcome variable (either responded or not responded). All additional parameters (hyperparameters) in the model were optimized using tenfold cross validation. *Canonical Correlation Analysis* [21] was used to analyze relations between the phenotypic and metabolomic matrix. Linear combinations of variables from each source dataset produced canonical variables reducing dimension in the dataset using the mixOmics package in R [22]. Two out of the 29 patients were unmedicated and were therefore not included in the Random Forest and Partial Least-Squares Discriminant Analysis (PLS-DA). For the selection of the metabolites to input in the PLS-DA we used variable importance projection (VIP) ranking; we considered VIP > 1.5 to be significant based on our previous research [23, 24].

The unsupervised methods applied to find patterns in the data set were *k*-means clustering and Principal Component Analysis (PCA). *K*-means clustering is a partition-based method that divides the samples or metabolites into subclusters that share similarities and are dissimilar to the objects belonging to another cluster. PCA minimizes the dimensionality of a data set consisting of many variables that are moderately or strongly associated with one another while maintaining as much variety as possible. The same is achieved by transforming the original variables into a new set of orthogonal variables known as the principal components (PCs) or dimensions, which preserve the original variation of the data sets. In this manner, the first principal component (PC1) preserves the greatest amount of variation from the original components. The eigenvectors of a covariance matrix serve as the major components, and as such, they are orthogonal.

We used the Pearson correlation (r) method to find associations between quantitative metabolites in this analysis. For the association between ketamine response and metabolites the Wilcoxon test was used. For both tests a p-value < 0.05 was considered significant.

## Results

For this pilot study, twenty-nine patients with treatment-resistant depression were selected from the chart review. Baseline characteristics of the patients and scores of the self-administered questionnaires are presented in Table 1. Data on the primary medication used (mood stabilizers: lithium and anticonvulsants, benzodiazepines, opioids, stimulants, antidepressants) and subsequent use of and response to ketamine or ketamine and rapamycin in combination are also indicated (Table 1).

**Table 1:** Baseline characteristics of the 29 patients with treatment-resistant depression

Variable	n (%)	Mean (SD)	Median	Range, min–max
Gender, male	12 (41.4%)			
Age, in years		40.6 (15.4)	40	12–65
BMI^a^, in kg/m^2^		28.4 (9.1)	28.3	15.4–61.2
Bipolar diagnosis, n (%)	6 (21%)			
Pain diagnosis, n (%)	13 (45%)			
Medication use				
Antidepressant	6 (21%)			
Stimulant	12 (41%)			
Benzodiazepine	11 (38%)			
Lithium	6 (21%)			
Opioids	10 (34%)			
Secondary medication				
Ketamine exposure^b^	21 (75%)			
Ketamine response^b^	9 (32%)			
Rapamycin/ketamine				
Combination exposure^b^	6 (21%)			
Rapamycin/ketamine				
Combination response^b^	3 (11%)			
Clinical checklist scores				
WHODAS^c^		18.4 (10.3)	19	1–37
PHQ-9^c^		12.3 (7.0)	12	2–27
Suicide^c,d^		0.4 (0.8)	0	0–3
GAD-7^c^		10.7 (6.8)	11	0–21
KSP-6^c^		15.3 (4.7)	16	6–24
AQ^e^		21.7 (9.5)	22	5–45

BMI, body mass index; GAD-7, 7-item Generalized Anxiety Disorder; KSP-6, 6-item Karolinska Scales of Personality; max, maximum; min, minimum; n, number; PHQ-9, 9-item Patient Health Questionnaire; SD, standard deviation; WHODAS, World Health Organization Disability Assessment Schedule; SD, standard deviation

^a^In 27 patients. ^b^In 28 patients. ^c^In 25 patients. ^d^Score derived from the suicidal thinking subscale number 9 of the PHQ-9. ^e^In 23 patients

### CSF metabolomic profile and 5-MTHF

The Metabolon analysis provided 300 individual metabolite values derived by comparing the data to reference data in an internal database (the raw data are deposited here: https://doi.org/10.6084/m9.figshare.21210656.v1). The 300 metabolites were reduced to the 151 metabolites that were present in all 29 samples (Supplemental Table S1) to avoid statistical issues associated with missing values. In addition, 5-MTHF was measured in all CSF samples. The mean 5-MTHF detected was 58.2 nmol/L (SD15.4), ranging from 29 to 85 nmol/L, and with a median value of 55 nmol/L.

### Metabolomic dimension reduction

We have used *k*-means clustering and number of optimum clusters defined by the silhouette criteria (Supplemental Figure S1A). The variations explained by the first 10 Principal components in decreasing order were visualized by a scree plot (Supplemental Figure S1B).

**Fig. 1:**
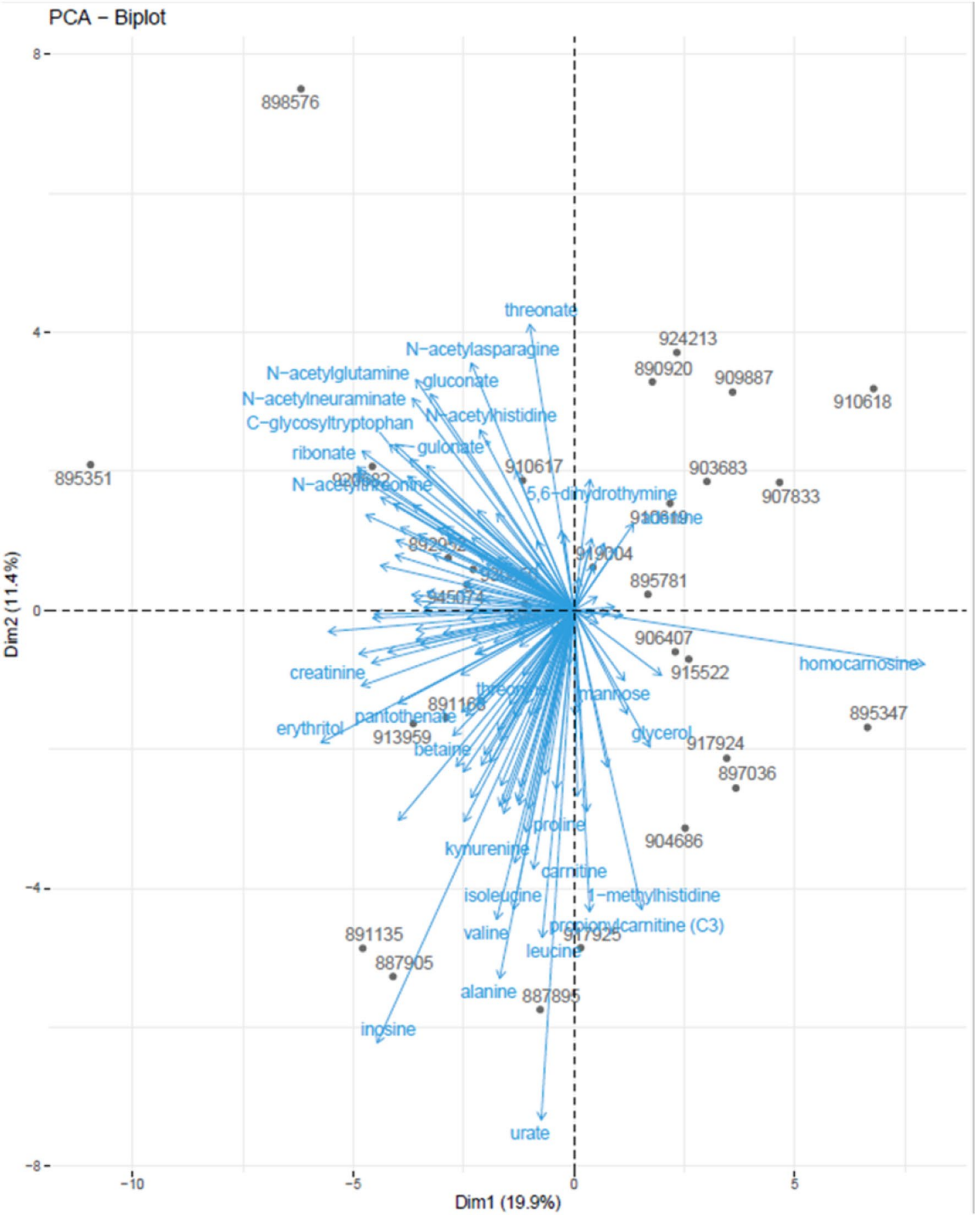
Principal component analysis of metabolites. The 151 metabolites are indicated in blue. Individual patients (n = 28) are indicated by a black dot and a patient number

A cluster plot of the 151 metabolites revealed the two main clusters. Individual metabolites separated from the central clusters differently, depending on statistical technique. Cluster analysis revealed the presence of homocarnosine and 2-hydroxyisobutyrate on the edges of cluster 2, with creatinine, fructose, trigonelline, urate, and propionylcarnitine (C3) on the edges of cluster 1 (Supplemental Figure S2). PCA analysis revealed and high projections of homocarnosine and creatinine on PC1 and very high projections of branched-chain amino acids (valine, leucine, isoleucine), C3, and urate on PC2 (Fig. 1).

In the 2-dimensional PCA scatterplot with 28 of the 29 patients (one patient was removed due to outliers in the PCA analysis), a non-random distribution of patients was noted. In quadrant IV (the upper left quadrant), patients 895351 and 898576 had minimal nearest neighbors relative to the other 27 samples (Fig. 1).

### Clinical data dimension reduction

**Fig. 2:**
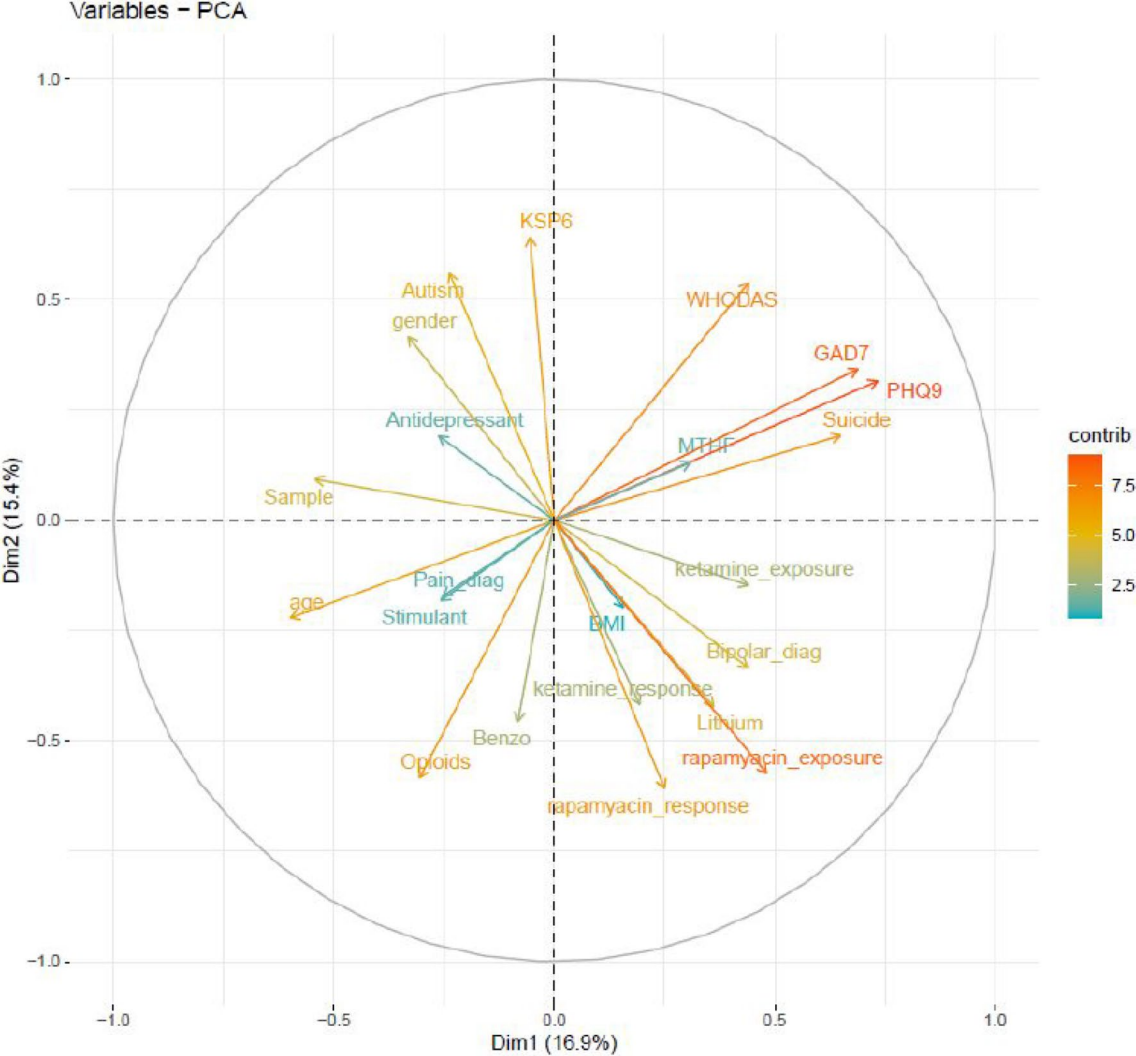
Principal component analysis of demographic and clinical data. BMI, body mass index; GAD-7, 7-item Generalized Anxiety Disorder; KSP-6, 6-item Karolinska Scales of Personality; PHQ-9, 9-item Patient Health Questionnaire; WHODAS, World Health Organization Disability Assessment Schedule; 5-MTHF, 5-methyltetrahydrofolate. Data from 28 patients were used in the PCA

Comorbidity in treatment-resistant depression is the rule rather than the exception. PCA analysis revealed that 32% of the variance in patient symptoms checklists, medication response, and demographic variables could be described by two PCs (Fig. 2). PC1 accounted for 16.9% of the variance and was defined by age and baseline mixed depression (PHQ-9) and anxiety (GAD-7) scores. PC2 accounted for 15.4% of the variance and was defined by depression (KSP-6 score), autism (AQ score) and male gender, inversely correlating with bipolar disorder, lithium treatment, ketamine response, and BMI. Note that the vectors for PHQ-9 score and lifetime bipolar diagnosis are almost perfectly orthogonal (Fig. 2). Figure 2 suggests that the 5-MTHF vector very closely aligns with the baseline PHQ-9 and GAD-7 vectors, which means that 5-MTHF correlated with PHQ9 (depression) and GAD-7 (anxiety) scores. In addition, the 5-MTHF vector was largely orthogonal to the ketamine response vector, indicating that 5-MTHF was not correlated with ketamine treatment response and thus not correlated with treatment-resistant depression. So, 5-MTHF was higher in patients with depression or anxiety, but not different according to treatment response.

### Associating phenotype and metabolite relationships

Mapping individual metabolites to a reduced dimensionality phenotype distribution revealed multiple metabolites of interest depending on Pearson correlation coefficients (r). At r = 0.6 stringency, the KSP-6 scores and rapamycin exposure overlapped strongly with PC2, while palmitoyl sphingomyelin (M120) and trimethylamine N-oxide (M143) overlapped strongly with PC1 (Supplemental Figure S3A).

At r = 0.5 stringency, additional overlaps appeared on both dimensions. Ethylmalonate (M85), autism (AQ score), rapamycin response, and lithium use loaded on PC2. Homocarnosine (M97), age, depression (PHQ-7 sore), and anxiety (GAD-7 score) loaded on PC1 (Figure S3B). At a lower level of stringency, r = 0.4, diagonal metabolites became more frequent, and clinical interpretation became more complicated (Figure S3C).

### Random forest regression results

Age accounted for 21.5% of the variance in the metabolome. We ranked the metabolites based on a random forest regression model that revealed the largest contributing monovariate metabolites were homocarnosine and 4-acetamidobutanoate. These two metabolites are both known to be related to gamma-aminobutyric acid (GABA) metabolism (Supplemental Figure S4).

### Ketamine response dimension reduction

A random forest modeling analysis revealed that the metabolites associated with the largest effect on clinical response to ketamine treatment were 2-hydroxyisobutyrate and n-acetylvaline. The metabolite 2-hydroxyisobutyrate was higher in the ketamine response class (test statistic: − 2.16; p = 0.058; not significant) (Supplemental Figure S5A) and n-acetylvaline was lower in the ketamine response class (test statistic: 2.06; p = 0.045; significant) (Supplemental Figure S5B).

Partial Least-Squares Discriminant Analysis (PLS-DA) also suggested a substantial loading for 2-hydroxybutyrate among the metabolites, with elevated variable importance projection (VIP) scores (Supplemental Figure S6). We selected the top four metabolites based on VIP scores (VIP > 1.5): glycerol, succinate, uridine, and 2-hydroxybutyrate/2-hydroxyisobutyrate. When we modeled only with those selected features (i.e., glycerol, succinate, uridine, and 2-hydroxybutyrate/2-hydroxyisobutyrate) the results were R2(X) 27.9%, R2(Y) 42.7 and Q2: 0.262. The model was not significant using Cross Validation ANalysis Of Variance (CV ANOVA). Multivariate gradient mapping suggested preferential responsiveness to ketamine treatment at r = 0.3 stringency in patients that were heavy, with low AQ scores, female, and previously treated for bipolar disorder.

## Discussion

In this pilot study, we retrospectively analyzed the CSF metabolome of 29 people with treatment-resistant depression to determine associations between metabolomic and phenotypic variation. Of the 300 metabolites analyzed, we identified 151 metabolites that were present in the CSF of all 29 individuals. Two dimensions were discovered in the CSF metabolome. These dimensions highly correlated with commonly used clinical data. Individual item metabolite analysis also suggested pathways possibly associated with ketamine response and possibly other medication treatments.

PC1 in the PCA analysis for the metabolic dimension reduction did not replicate a previous study suggesting central folate deficiency is a common cause of treatment-resistant depression [6]. Given this targeted univariate CSF analysis suggested a high correlation between depression and high 5-MTHF levels, inconsistent with l-methylfolate FDA approval as an augmentation agent, we thus found no evidence for 5-MTHF deficiency associated with treatment-resistant depression. The correlation was even in the opposite direction. We also observed that two of the 29 patients with 5-MTHF levels below the 5% laboratory population estimate showed no response to folinic acid supplementation.

PC1, given high overlaps between age and homocarnosine, may also estimate integral gain of function population variation in astroglial differentiation. Elevations in homocarnosine, a metabolite produced mainly in astroglia [29], are known to be strongly correlated with age. In mice, age is associated with increased phenotypic conversion into the A1 pro-inflammatory astrocyte with associated downregulation of mitochondrial and antioxidant defense genes [30]. An underlying associated activation of microglia may drive a coupled inflammatory phenotype, perhaps consistent with elevated palmitoyl sphingomyelin on this axis, suggestive of increased peroxisomal biogenesis and/or activity [31]. These data suggested that the PC1 gradient may correspond to the brain's equilibrium distribution of the A1 and A2 astroglia.

PC2 in the PCA analysis for the metabolic dimension reduction had high projections of branched-chain amino acids, propionylcarnitine (C3), and urate, and presumably overlaps with the population integral gain of function variation in nitrogen utilization and/or mitophagy programs. Activity in these pathways may causally associate with specific clinical syndromes or medication selection.

In the clinical data dimension reduction, the correlation between "objective" CSF PC2 and the "subjective" KSP-6 that estimated brain mitochondrial activity was surprisingly high (r = 0.7) in the diverse clinical sample. This correlation suggests muscle and CSF pathophysiology in self-reported "sickness behavior" is essentially the same. Items of the KSP-6 were historically selected to maximally correlate with alpha-ketoglutarate consumption via complex 1 (C1) in fresh muscle biopsy [13]. Although elevated scores on the KSP-6 have been reported to correlate with pathological heteroplasmic variability in families with primary mitochondrial disorder, untargeted muscle biopsy suggests decreased C1 activity is related more to decreased mitochondrial mass in muscle samples from patients with syndromic chronic fatigue syndrome [25].

Equilibrium mitochondrial mass is a function of both baseline mitophagy and mitochondrial biogenesis. The relative contribution of these processes cannot be disentangled by an aggregate statistic. However, a primary defect in mitochondrial biogenesis was inconsistent with one data point in the sample: patient 895781 that projects minimally onto PC1. This patient has a classical syndrome of treatment-resistant post-traumatic epilepsy and progressive dementia, mutations in the *TWNK* and *POLG* genes that drive mitochondrial biogenesis, serum medium-chain acylcarnitine elevation, and acute valproic acid hepatotoxicity.

The correlation we found in the clinical data dimension reduction between PC2 and autism was of significant interest (r = 0.5) in this diverse clinical sample. Recent data suggested that maximal C1 activity in fibroblasts of children with autism spectrum disorder was impaired in two disjoint ways with diverging phenotypes associated with the larger aggregate autism disorder [26]. We suspect that our community sample is most consistent with the phenotype marked by impaired social cognition, social motivation, and social withdrawal rather than stereotypy. This phenotype is, in turn, associated with mitochondrial uncoupling and elevated respiratory rates, possibly related to excessive inhibition of genes related to mitochondrial quality control by TOR1/SK61. A similar pattern was reported in patients with chronic fatigue syndrome, which was also marked by an elevated rate of complex IV activity [25].

The correlation between PC2 and bipolar disorder/lithium use we found was of moderate interest (r = 0.4) in this diverse clinical sample. Of note, in the Lithium Treatment-Moderate dose Use Study (LiTMUS), that analyzed blood of lithium responders and non-responders, some of the largest overlaps between lithium response and non-response were defined by increased expression of genes involved in neutral cell-surface amino acid transport (*SLC1A5*, *SLC6A9,* and *SLC7A5*) and glutamine synthesis (*GLUL*) with implications for baseline mTOR activity in terms of nitrogen-sensing dependent activity [27]. Previous metabolomic analysis of CSF revealed increased isocitrate correlates with a diagnosis of bipolar disorder in males [5]. There may also be a modest overlap between the insensitivity to anesthesia observed in individuals with genetic defects of C1 and insensitivity to sedation and ataxia from benzodiazepine use in bipolar patients [28]

Although multiple correlations were observed between response to standard treatments, we here report on ketamine, given its remarkable efficacy in treatment-resistant depression. Ketamine, alone or in conjunction with its recently described augmentation agent rapamycin, has profound effects on TOR activity both in terms of specific biochemical changes and corresponding phenotypic effect, e.g., acute synaptic growth 1 h after infusion [29]. Although the phenotypic ketamine response projected moderately on the PC2 axis, consistent with MTOR function in control of mitophagy and intracellular nitrogen utilization, systematized item metabolite analysis also directs attention to the trend of elevated 2-hydroxyisobutyrate. Acylation of this metabolite selectively targets glycolytic enzymes and is essential for cell survival in response to glucose rather than glutamine restriction [30].

The metabolome associated with clinical ketamine response we found was reminiscent of changes in metabolomic profiles in healthy young males exercising in anoxic conditions [31]. In that study, in the context of inadequate substrate availability, oxygen/cellular NADH ratios increased with a resultant increase in glutathione synthesis (2-hydroxybutyrate byproduct) and decreased reliance on carbohydrate energy stores (glucose utilization) relative to alternative energy stores (branched-chain amino acids) [31]. The effect of baseline TOR1/TOR2 activity on the response variance observed in the highly screened "healthy" males in this study has not been studied. In our results, high 2-hydroxybutyrate also correlated with lithium use, confirming the previous discussion of lithium responsiveness that was highly associated with changes in neutral amino acid transport.

Comparison with a recent report describing principal metabolomic components in the plasma and the CSF in nine "normal" volunteers following a single IV ketamine infusion may provide additional context [32]. In that study, 87 CSF metabolites were clustered into four components, with only PC2 exhibiting consistent changes over 28 h. Serum and CSF loadings were in opposite directions for leucine, isoleucine, tryptophan, methionine, and tyrosine. The authors discussed their findings in terms of TOR activation, IDO effects on tryptophan metabolism with possible implications for NAD homeostasis, sphingolipid signaling, and rate-limited precursors for cardiolipin synthesis [32]. The applicability of that study to our patient group with chronic daily use is potentially limited by referral bias. In clinical practice, chronic daily high-dose ketamine treatment is most commonly used for chronic pain, while pulsed ketamine treatment is most effective for affective disorders. An elevated prevalence of patients maintained on opioids in our sample relative to standard affective disorder protocols in academic settings is noted. Although the genetics of chronic pain and depression were found to be highly correlated in a large-scale genome-wide association study [33], differential phenotypic expression implies divergent pathophysiology. Affective disorders response may be associated with an anabolic activation of microglial brain-derived neurotrophic factor secretion in prefrontal cortex [34]. Decreased concentration of branched-chain amino acids in CSF relative to serum would be expected, consistent with increased essential amino acid flux similar to what has been observed with use-dependent muscle hypertrophy [35] and perhaps with blood gene expression associated with lithium response [27]. Treatment of chronic pain with ketamine, in contrast, marked by classical subjective inflammatory symptoms, downregulates an observed chronic inflammatory astroglial phenotype [36], and possibly changes basal M2 microglial synaptic pruning modulated by nuclear neuronal NFATc4 expression and subsequent IL-4 secretion [37, 38].

Further opportunities for the use of CSF metabolome analysis abound. A reliable age marker in the CSF, "an aging clock," may allow for selection among the numerous FDA-approved drugs which increase lifespan in animal models (lithium, rapamycin, tamoxifen, metformin) for primary treatment of mood disorders, secondary prophylaxis in acutely stabilized depression, and primary prophylaxis in case of a family history of dementia [39]. Repeated measures could verify efficacy in each patient and suggest dose modulation, similar to protocols used in diabetes glucose and lipid assessment. If replicated, reliable fuel-switching markers in the CSF may also allow for better patient stratification in both aggregate trials and N = 1 clinical practice. Public databases of ketamine response coupled with a unique CSF profile (e.g., wapainmanage.org) could also be searched by patients, their physicians, or specialty disease non-profit organizations to allow for off-label, inexpensive community-driven hypothesis testing under emerging Right-to-Try federal and state laws.

In our experience, patient acceptance of CSF metabolome analysis was very high. By definition, patients in close proximity on the 2-D metabolomic graph are biochemical relatives, similar to the genetic kinships recently discovered via personal direct-to-consumer genetic testing or online support groups for patients with defined single gene defects. Close proximity in a 2-D vector space accounting for 35% of the variance implies a closer physiological resemblance than a second-degree relative as inferred from simple Mendelian genetics. Many of our patients have a tremendous interest in either meeting their nearest "metabolomic buddy" or reviewing the redacted clinical history of their nearest "metabolomic buddy" with their provider. Motivated by the clinical history of their nearest metabolomic neighbors, we have seen hopeless patients successfully select and subsequently respond to high number-needed-to-treat (NNT) augmentation agents.

This study's major strengths are, paradoxically, its small sample size, unstructured hypothesis-free patient selection, and exploration of biomarkers of chronic TOR modulators (ketamine alone or in combination with rapamycin). In this context, observed effect sizes were large enough to have clinically meaningful input into patient management in contrast to large studies (N = 60–100), which produce statistically significant results, but the NNT is in the order of 5–10. Generalizability of results is statistically implied by dimensional markers of pathophysiology rather than categorical and thus inherently underpowered binary patient groups, e.g., unmedicated depressed vs. remitted depressed (bipolar) groups described in previous metabolomic studies [40]. Reference to so-called "normal controls" is highly questionable in terms of population central tendency estimation, even in our community practice we have observed positive recruitment bias for autism spectrum defined not only by dimensional symptom checklists currently in development but also by delayed and self-limited fecundity not typically seen in mood disorder [41]. This study is also the only current source of clinical information for CSF biomarkers associated with chronic ketamine use alone and combined with rapamycin.

This study has several limitations. First, the proximal sources of the metabolites are unknown. These sources are potentially affected by blood–brain barrier kinetics with leakage of systemic metabolites into the CSF pool, different caudal vs. ventral neuroaxis physiology, cell type source, and patient variability in baseline diet and exercise. Second, of many CSF metabolites, minimal information is available to interpret their effect because the physiology associated with their variability has not yet been studied in animal models or people with genetic gain-of-function or loss-of-function variants. Third, CSF metabolites were not analyzed in a control group of patients with depression that is not treatment-resistant, or in a control group of people without signs of depression. Fourth, the metabolites were measured once in these patients, so whether the measurements are incidental, due to the clinical diagnosis, or due to the treatment is unknown. Future studies should include metabolome analysis before, during, and after treatment.

Even assuming the above limitations are met, the field will likely continue to struggle with the technical problems of using linear statistical models to analyze metabolites with individually widely divergent non-normal distributions. By convention, the z-score transform is typically used to force some degree of linearity. This convention, however, has many known limitations. First, in individual samples, large + z-scores will be over-represented relative to -z-scores given sampling bias associated with limits of detection of metabolites at low concentrations. Second, sampling bias in the reference database generating z-scores can potentially influence the distribution of the patient z-score distribution; this bias need not be linear and may not easily be corrected by standard regression models. Figure 1 provides an excellent example of this problem. Although a linear model is extremely successful in dimensionality reduction, the density asymmetry on PCA1 is visually striking. The number of large amplitude individual metabolites (visible) is not equivalent on the left and right halves of the x-axis, although by PCA definition the total weights of integral small (invisible) and large (visible) metabolite amplitudes must be the same. This density asymmetry may have physiological meaning and serves as a useful reminder of the limitations of linear statistical models. The computational, distinct from the physiological, implications of a sparse vs. dense dataset when incorporated into predictive clinical tools is currently an area of intense inquiry in large language model development [42].

In conclusion, we found that complex treatment-resistant depression can be mapped onto a 2-dimensional pathophysiological domain based on CSF metabolome data. The first dimension's components may reflect the involvement of age-related astroglial differentiation programs, while the second dimension's components may reflect the involvement of nitrogen sensing or mitophagy pathways. The results of this pilot study may have implications for combinatorial medication selection for depression subtypes.

Application of these data compression algorithms into standard clinical practice will be challenging. Clinicians will need to manage a more complicated diagnostic landscape than the current DSM-5 criteria. A Boolean description of a patient’s depression phenotype, bipolar vs. unipolar, will need to be replaced by a vector of principal pathophysiological components. Holding this vector in working memory while interacting with the individual patients is cognitively more strenuous in our experience. However, it does not escape our notice that this diagnostic complexity more faithfully represents the polypharmacy that is increasingly successfully utilized in the community and in current regulatory protocols.

### Supplementary Information

Below is the link to the electronic supplementary material.

**Supplementary file 1:** **Supplemental Table S1**. List of metabolites analyzed. **Supplemental Figure S1**. Optimal number of dimensions. A) based on k-means clustering B) based on PCA, visualized with a scree plot. **Supplemental Figure S2**. Cluster plot of the 151 metabolites. Two clusters can be distinguished in the cluster plot. The pink circles represent the metabolites of cluster 1, the blue triangles represent the metabolites of cluster 2. The centroid of each cluster is represented by a slightly larger shape. **Supplemental Figure S3**. Mapping metabolites to a reduced dimensionality phenotype distribution. A) at r = 0.6 stringency, B) at r = 0.5 stringency, C) r = at 0.4 stringency. GAD-7, 7-item Generalized Anxiety Disorder; KSP-6, 6-item Karolinska Scales of Personality; PHQ-9, 9-item Patient Health Questionnaire; WHODAS, World Health Organization Disability Assessment Schedule; 5-MTHF, 5-methyltetrahydrofolate. **Supplemental Figure S4**. Using age as a response variable and ranking of the associated metabolites based on the % IncMSE. IncMSE, increase in mean squared error of predictions. **Supplemental Figure S5**. Random Forest modelling. A) relation of hydroxyisobutyrate with ketamine response, B) relation of acetylvaline with ketamine respons**Supplemental Figure S6**. VIP scores Partial Least-Squares Discriminant Analysis (PLS-DA) suggested a substantial loading for 2-hydroxybutyrate among the metabolites, with elevated VIP scores. 1 = ketamine responder, 0 = ketamine non-responder. BMI, body mass index. VIP, variable importance projection.

## Data Availability

The data that support the findings of this study are available upon reasonable request from the corresponding author.
