# Supplementary material for: Cerebrospinal fluid metabolomes of treatment-resistant depression subtypes and ketamine response: a pilot study
Source: Discov Ment Health. 2024 Apr 17;4(1):12. doi: 10.1007/s44192-024-00066-5 (PMC11024073; doi:10.1007/s44192-024-00066-5)
Supplement: Supplementary file 1 — Supplemental Table S1. List of metabolites analyzed. Supplemental Figure S1. Optimal number of dimensions. A) based on k-means clustering B) based on PCA, visualized with a scree plot. Supplemental Figure S2. Cluster plot of the 151 metabolites. Two clusters can be distinguished in the cluster plot. The pink circles represent the metabolites of cluster 1, the blue triangles represent the metabolites of cluster 2. The centroid of each cluster is represented by a slightly larger shape. Supplemental Figure S3. Mapping metabolites to a reduced dimensionality phenotype distribution. A) at r = 0.6 stringency, B) at r = 0.5 stringency, C) r = at 0.4 stringency. GAD-7, 7-item Generalized Anxiety Disorder; KSP-6, 6-item Karolinska Scales of Personality; PHQ-9, 9-item Patient Health Questionnaire; WHODAS, World Health Organization Disability Assessment Schedule; 5-MTHF, 5-methyltetrahydrofolate. Supplemental Figure S4. Using age as a response variable and ranking of the associated metabolites based on the % IncMSE. IncMSE, increase in mean squared error of predictions. Supplemental Figure S5. Random Forest modelling. A) relation of hydroxyisobutyrate with ketamine response, B) relation of acetylvaline with ketamine responsSupplemental Figure S6. VIP scores Partial Least-Squares Discriminant Analysis (PLS-DA) suggested a substantial loading for 2-hydroxybutyrate among the metabolites, with elevated VIP scores. 1 = ketamine responder, 0 = ketamine non-responder. BMI, body mass index. VIP, variable importance projection. [file 44192_2024_66_MOESM1_ESM.docx]

# **Supplementary materials**

**Cerebrospinal fluid metabolomes of treatment-resistant depression subtypes and ketamine response: a pilot study**

Jon Berner, MD PhD^1^ and Animesh Acharjee, PhD^2,3,4^

^1^Woodinville Psychiatric Associates, Woodinville, WA, USA

^2^Institute of Cancer and Genomics Sciences, University of Birmingham, UK

^3^Institute of Translational Medicine, University Hospitals Birmingham NHS Foundation Trust, Birmingham, UK

^4^MRC Health Data Research UK (HDR UK), London, UK

**Corresponding author:** Jon Berner, MD PhD

Woodinville Psychiatric Associates, 18500 156th Ave NE #100, Woodinville, WA 98072, United States. Tel: +1-425-481-0429. Email: [jonbernermd@gmail.com](mailto:jonbernermd@gmail.com)

ORCID: 0000-0003-3117-6482

## **Supplemental Table S1 List of metabolites analyzed**

| **Number** | **Name** |
| --- | --- |
| M1 | 1,2-dipalmitoyl-GPC (16:0/16:0) |
| M2 | 1,5-anhydroglucitol (1,5-AG) |
| M3 | 1-methylhistidine |
| M4 | 1-methylnicotinamide |
| M5 | 1-myristoyl-2-palmitoyl-GPC (14:0/16:0) |
| M6 | 1-oleoyl-2-linoleoyl-GPC (18:1/18:2) |
| M7 | 1-oleoyl-GPC (18:1) |
| M8 | 1-palmitoyl-2-arachidonoyl-GPC (16:0/20:4n6) |
| M9 | 1-palmitoyl-2-dihomo-linolenoyl-GPC (16:0/20:3n3 or 6) |
| M10 | 1-palmitoyl-2-linoleoyl-GPC (16:0/18:2) |
| M11 | 1-palmitoyl-2-oleoyl-GPC (16:0/18:1) |
| M12 | 1-palmitoyl-2-palmitoleoyl-GPC (16:0/16:1) |
| M13 | 1-palmitoyl-GPC (16:0) |
| M14 | 1-stearoyl-2-arachidonoyl-GPC (18:0/20:4) |
| M15 | 1-stearoyl-2-linoleoyl-GPC (18:0/18:2) |
| M16 | 1-stearoyl-2-oleoyl-GPC (18:0/18:1) |
| M17 | 2-hydroxy-3-methylvalerate |
| M18 | 2-hydroxybutyrate/2-hydroxyisobutyrate |
| M19 | 2-piperidinone |
| M20 | 3-(4-hydroxyphenyl)lactate |
| M21 | 3-hydroxy-3-methylglutarate |
| M22 | 3-hydroxybutyrate (BHBA) |
| M23 | 3-hydroxyisobutyrate |
| M24 | 3-methyl-2-oxovalerate |
| M25 | 3-methylglutaconate |
| M26 | 3-methylglutarylcarnitine (2) |
| M27 | 4-acetamidobutanoate |
| M28 | 4-methyl-2-oxopentanoate |
| M29 | 5,6-dihydrothymine |
| M30 | 5-methylthioadenosine (MTA) |
| M31 | 5-methyluridine (ribothymidine) |
| M32 | 5-oxoproline |
| M33 | 6-oxopiperidine-2-carboxylate |
| M34 | 7-alpha-hydroxy-3-oxo-4-cholestenoate (7-Hoca) |
| M35 | 7-methylguanine |
| M36 | C-glycosyltryptophan |
| M37 | N-acetyl-aspartyl-glutamate (NAAG) |
| M38 | N-acetyl-beta-alanine |
| M39 | N-acetylalanine |
| M40 | N-acetylarginine |
| M41 | N-acetylasparagine |
| M42 | N-acetylaspartate (NAA) |
| M43 | N-acetylglutamate |
| M44 | N-acetylglutamine |
| M45 | N-acetylglycine |
| M46 | N-acetylhistidine |
| M47 | N-acetylmethionine |
| M48 | N-acetylneuraminate |
| M49 | N-acetylputrescine |
| M50 | N-acetylserine |
| M51 | N-acetyltaurine |
| M52 | N-acetylthreonine |
| M53 | N-acetylvaline |
| M54 | N-formylmethionine |
| M55 | N1-methyladenosine |
| M56 | N2,N2-dimethylguanosine |
| M57 | N6,N6,N6-trimethyllysine |
| M58 | O-sulfo-L-tyrosine |
| M59 | acetylcarnitine (C2) |
| M60 | adenine |
| M61 | alanine |
| M62 | alpha-hydroxyisocaproate |
| M63 | alpha-hydroxyisovalerate |
| M64 | arabitol/xylitol |
| M65 | arabonate/xylonate |
| M66 | arginine |
| M67 | asparagine |
| M68 | beta-hydroxyisovalerate |
| M69 | beta-hydroxyisovaleroylcarnitine |
| M70 | betaine |
| M71 | carboxyethyl-GABA |
| M72 | carnitine |
| M73 | choline |
| M74 | choline phosphate |
| M75 | citrulline |
| M76 | creatine |
| M77 | creatinine |
| M78 | cysteine |
| M79 | cytidine |
| M80 | deoxycarnitine |
| M81 | dimethylarginine (SDMA + ADMA) |
| M82 | dimethylglycine |
| M83 | erythritol |
| M84 | erythronate |
| M85 | ethylmalonate |
| M86 | fructose |
| M87 | gluconate |
| M88 | glucose |
| M89 | glucuronate |
| M90 | glutamate |
| M91 | glutamine |
| M92 | glycerol |
| M93 | glycine |
| M94 | gulonate |
| M95 | histidine |
| M96 | homoarginine |
| M97 | homocarnosine |
| M98 | homovanillate (HVA) |
| M99 | hypoxanthine |
| M100 | inosine |
| M101 | isobutyrylcarnitine (C4) |
| M102 | isoleucine |
| M103 | kynurenine |
| M104 | lactate |
| M105 | leucine |
| M106 | lysine |
| M107 | malate |
| M108 | malonate |
| M109 | mannitol/sorbitol |
| M110 | mannose |
| M111 | methionine |
| M112 | methionine sulfone |
| M113 | methylmalonate (MMA) |
| M114 | methylsuccinoylcarnitine |
| M115 | myo-inositol |
| M116 | nicotinamide riboside |
| M117 | ornithine |
| M118 | orotate |
| M119 | orotidine |
| M120 | palmitoyl sphingomyelin (d18:1/16:0) |
| M121 | pantothenate |
| M122 | phenol sulfate |
| M123 | phenylacetylglutamine |
| M124 | phenylalanine |
| M125 | pipecolate |
| M126 | proline |
| M127 | propionylcarnitine (C3) |
| M128 | pseudouridine |
| M129 | ribitol |
| M130 | ribonate |
| M131 | serine |
| M132 | sphingomyelin (d18:1/18:1, d18:2/18:0) |
| M133 | sphingomyelin (d18:2/16:0, d18:1/16:1) |
| M134 | stachydrine |
| M135 | stearoyl sphingomyelin (d18:1/18:0) |
| M136 | succinate |
| M137 | succinylcarnitine (C4-DC) |
| M138 | sulfate |
| M139 | taurine |
| M140 | threonate |
| M141 | threonine |
| M142 | trigonelline (N'-methylnicotinate) |
| M143 | trimethylamine N-oxide |
| M144 | tryptophan |
| M145 | tryptophan betaine |
| M146 | tyrosine |
| M147 | urate |
| M148 | urea |
| M149 | uridine |
| M150 | valine |
| M151 | xanthine |

## **Supplemental Figure S1 Optimal number of dimensions**

**A**) based on k-means clustering **B**) based on PCA, visualized with a scree plot


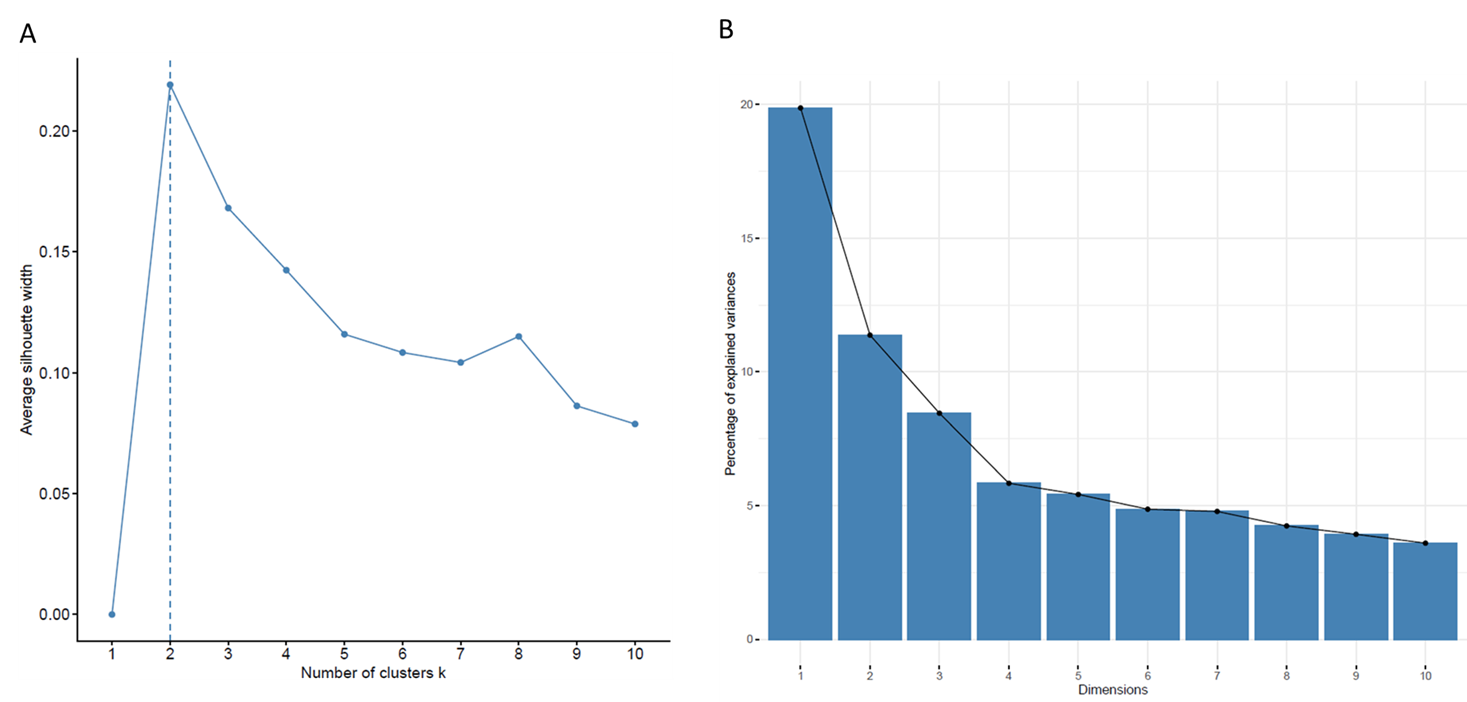


## **Supplemental Figure S2 Cluster plot of the 151 metabolites**

Two clusters can be distinguished in the cluster plot. The pink circles represent the metabolites of cluster 1, the blue triangles represent the metabolites of cluster 2. The centroid of each cluster is represented by a slightly larger shape.


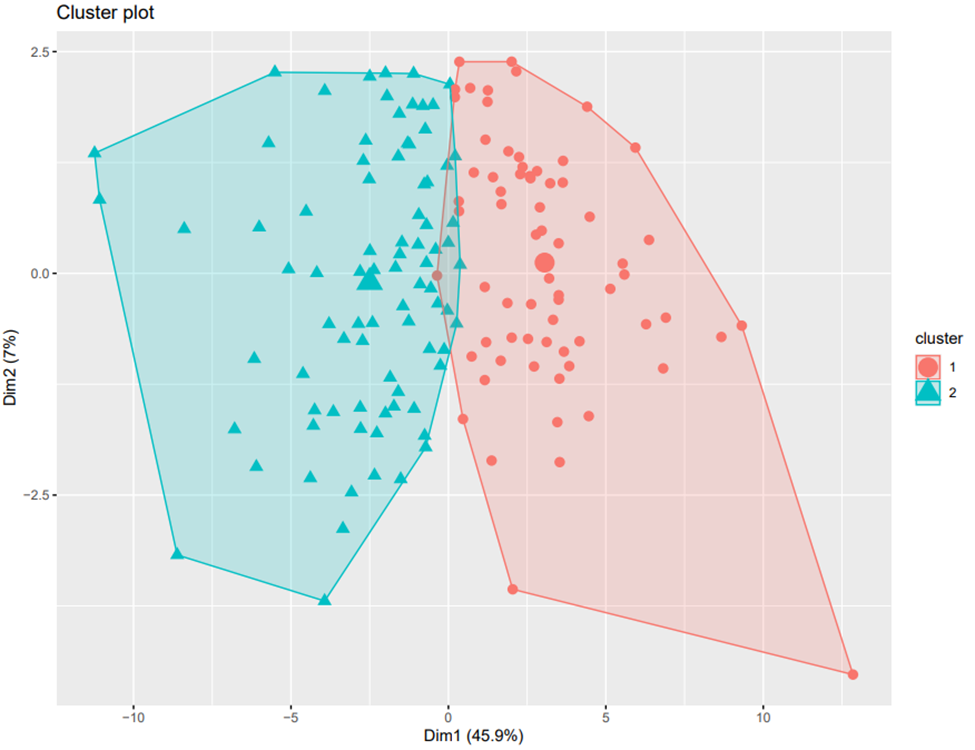


## **Supplemental Figure S3** **Mapping metabolites to a reduced dimensionality phenotype distribution**

A) at r = 0.6 stringency, B) at r = 0.5 stringency, C) r = at 0.4 stringency.

GAD-7, 7-item Generalized Anxiety Disorder; KSP-6, 6-item Karolinska Scales of Personality; PHQ-9, 9-item Patient Health Questionnaire; WHODAS, World Health Organization Disability Assessment Schedule; 5-MTHF, 5-methyltetrahydrofolate


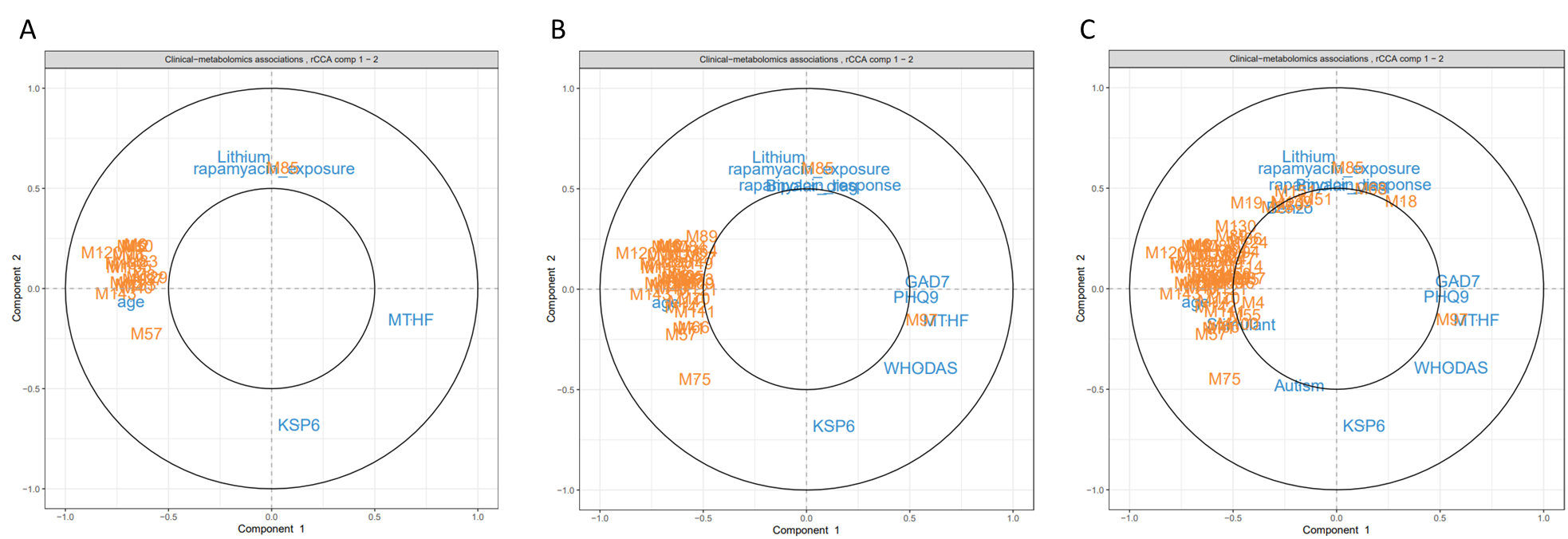


## **Supplemental Figure S4 Using** **age as a response variable and ranking of the associated metabolites based on the % IncMSE**

IncMSE, increase in mean squared error of predictions.

## **Supplemental Figure S5** **Random Forest modeling**

A) relation of hydroxyisobutyrate with ketamine response, B) relation of acetylvaline with ketamine response.


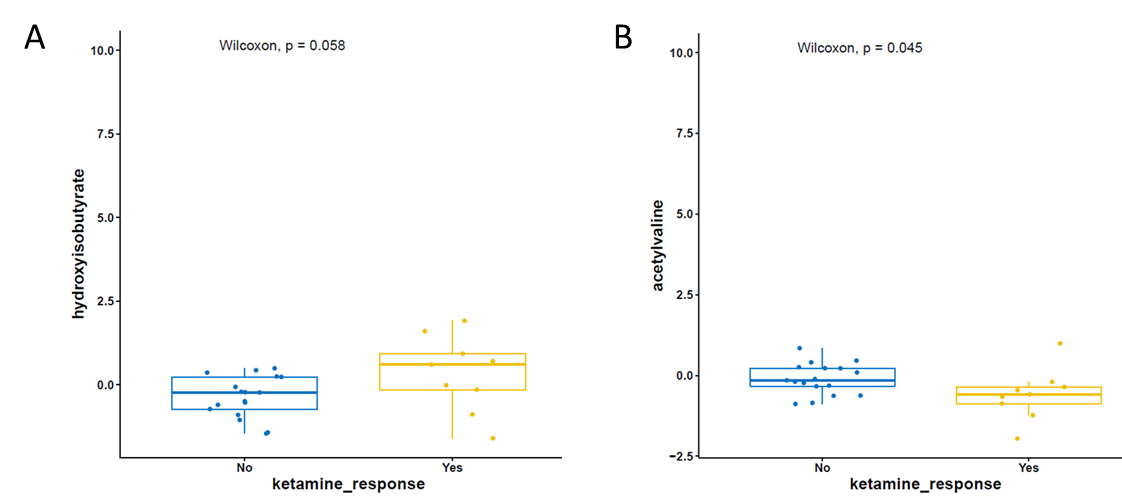


## **Supplemental** **Figure S6 VIP scores**

Partial Least-Squares Discriminant Analysis (PLS-DA) suggested a substantial loading for 2-hydroxybutyrate among the metabolites, with elevated VIP scores. 1 = ketamine responder, 0 = ketamine non-responder.

BMI, body mass index. VIP, variable importance projection.

**
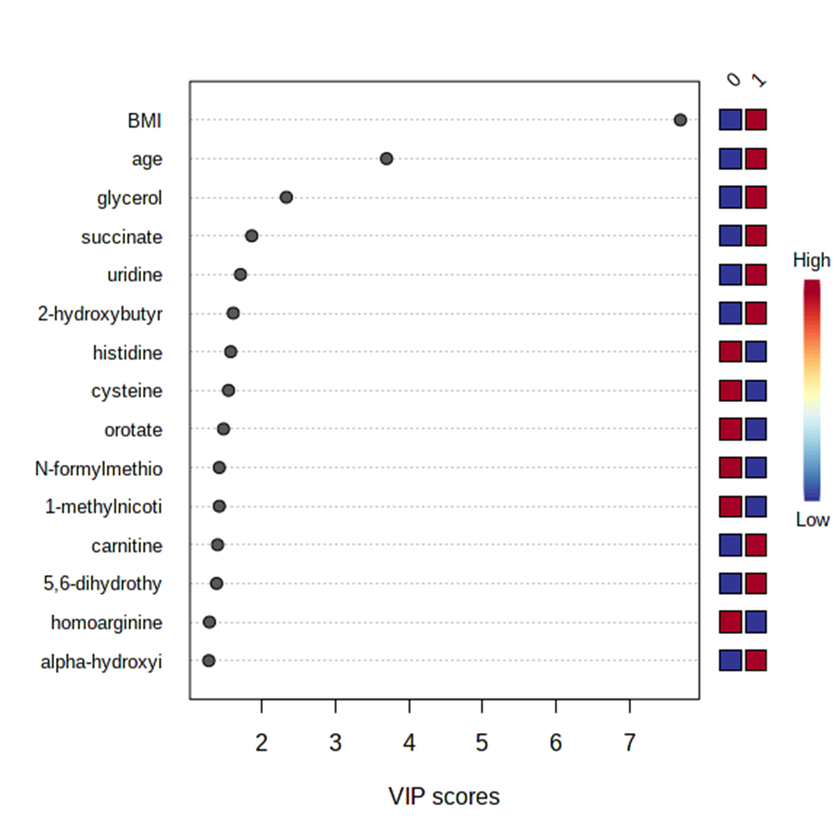
**
